# IRF4 downregulation improves sensitivity and endurance of CAR T cell functional capacities

**DOI:** 10.3389/fimmu.2023.1185618

**Published:** 2023-05-23

**Authors:** Dennis Christoph Harrer, Valerie Bezler, Jordan Hartley, Wolfgang Herr, Hinrich Abken

**Affiliations:** ^1^ Dept. Hematology and Medical Oncology, Clinic III Internal Medicine, University Hospital Regensburg, Regensburg, Germany; ^2^ Leibniz Institute for Immunotherapy, Div. Genetic Immunotherapy, Regensburg, Germany

**Keywords:** CAR, IRF4, exhaustion, sensitivity, tumor

## Abstract

Chimeric antigen receptor (CAR) modified T cells can induce complete remissions in patients with advanced hematological malignancies. Nevertheless, the efficacy is mostly transient and remains so far poor in the treatment of solid tumors. Crucial barriers to long-term CAR T cell success encompass loss of functional capacities known as “exhaustion”, among others. To extend CAR T cell functionality, we reduced interferon regulatory factor 4 (IRF4) levels in CAR T cells using a one-vector system encoding a specific short-hairpin (sh) RNA along with constitutive CAR expression. At baseline, CAR T cells with downregulated IRF4 showed equal cytotoxicity and cytokine release compared to conventional CAR T cells. However, under conditions of repetitive antigen encounter, IRF4^low^ CAR T cells displayed enhanced functionality with superior cancer cell control in the long-term compared with conventional CAR T cells. Mechanistically, the downregulation of IRF4 in CAR T cells resulted in prolonged functional capacities and upregulation of CD27. Moreover, IRF4^low^ CAR T cells were more sensitive to cancer cells with low levels of target antigen. Overall, IRF4 downregulation capacitates CAR T cells to recognize and respond to target cells with improved sensitivity and endurance.

## 1 Introduction

Chimeric antigen receptor (CAR) T cells evolved into a crucial pillar of cancer immunotherapy in recent years (1). Following long-lasting complete remissions in patients with advanced B-cell malignancies, regulatory authorities in the US and Europe issued approval for CAR T cell treatment in patients with refractory or relapsing acute lymphoblastic leukemia and specific lymphoma entities (2). While numerous clinical trials are successfully evaluating CAR T cell therapy in a wide variety of hematological malignancies, a sizable portion of patients does not benefit from CAR T cell therapy. Moreover, CAR T cell therapy of solid tumors showed poor results so far (3, 4). Given these insufficiencies, preclinical refinement of CAR T cell constructs is ongoing to improve and extend the power of CAR T cell therapy and to overcome T cell dysfunctionality upon repetitive target engagement (5, 6).

T cell dysfunctionality, commonly termed “exhaustion”, and entry into cell death are intrinsic barriers limiting T cell activation and finally therapeutic efficacy (7, 8). Hallmarks of T cell exhaustion include differentiation into the effector cell compartment, upregulation of inhibitory receptors, reduced proliferative capacity, lack of IL-2 production, as well as diminished cytotoxicity and reduced release of pro-inflammatory cytokines (9, 10). Based thereon, a plethora of efforts are currently undertaken to prevent or counteract CAR T cell exhaustion once the CAR recognizes its target. These efforts encompass the use of long-lived, self-renewing, multipotent T memory stem cells (TSCMs) (11, 12), the knockdown of inhibitory molecules (13–17), the engraftment of artificial IL-9 signaling (18), and the optimization of co-stimulatory signals to the CAR T cell (19–21). On the other hand, a growing body of evidence implies specific transcription factors, such as BLIMP-1 and TOX, in inducing and maintaining exhaustive phenotypes in CAR T cells (22, 23). Correspondingly, CAR T cells with knockdown of BLIMP-1 or TOX displayed a reduced propensity to exhaustion eventuating in an augmented CAR T cell functionality *in vitro* and superior CAR T cell performance in tumor bearing mice (24, 25).

More recently, the T cell receptor (TCR)-induced transcription factor interferon regulatory factor 4 (IRF4) is gaining attention in the context of T cell exhaustion. Seminal evidence linking IRF4 to T cell exhaustion was derived from mice with chronic lymphocytic choriomeningitis virus (LCMV) infection (26). In these mice, antigen-specific T cells expressed high IRF4 levels associated with upregulated inhibitory receptors, such as PD-1, repressed memory-associated regulators, like TCF1, and triggered metabolic stress reactions. The exhausted stage could be reversed by targeted decrease of IRF4 levels resulting in highly functional antigen-specific T cells with a memory-like phenotype (26). On the other hand, IRF4 was recently found to be upregulated in CARs with artificially high tonic signaling driving T cells into exhaustion (9). Overexpression of the transcription factor c-Jun abrogated T cell exhaustion in tonically signaling CARs. Remarkably, this process was accompanied by the downregulation of IRF4 and other exhaustion-associated genes regulated by IRF4 (9). Taken together, we see a crucial role of IRF4 in establishing and maintaining T cell exhaustion.

We here aimed at improving anti-cancer cell functionality of CAR T cells by reducing IRF4 levels. Given a physiological role for IRF4 in T cell biology, such as activation, expansion and functionality of CD8^+^ T cells, we did not seek to completely abrogate IRF4 expression in CAR T cells (27, 28), but rather substantially reduce IRF4 levels. With respect to manufacturing, we newly designed a one-vector system that encodes both the shRNA for reducing IRF4 levels and the CAR for constitutive expression. Under conditions of repetitive antigen stimulation, CAR T cells with downregulated IRF4 performed superior with respect to anti-tumor effector functions as compared to conventional CAR T cells. Mechanistically, we observed that the downregulation of IRF4 in CAR T cells resulted in prolonged functionality and upregulation of CD27. Moreover, such CAR T cells were capable of targeting otherwise neglected target cells with low antigen levels demonstrating a strategy to improve CAR T cell sensitivity towards cancer cells.

## 2 Materials and methods

### 2.1 Cells and reagents

Peripheral blood mononuclear cells (PBMCs) were obtained by Lymphoprep centrifugation (Axis-Shield, Oslo, Norway) of blood from healthy donors upon informed consent and approval by the institutional review board. Isolated PBMCs were cryopreserved and stored at -80°C until experimental use. T cells were maintained in RPMI 1640 medium, 1% GlutaMAX (Gibco, ThermoFisher, Waltham, MA, USA), 100 IU/mL penicillin, 100 µg/mL streptomycin (Pan-Biotech, Aidenbach, Germany), 2 mM HEPES (PAA, Palo Alto, CA, USA), and 10% (v/v) heat-inactivated fetal calf serum (Pan-Biotech, Aidenbach, Germany). 293T cells are human embryonic kidney cells that express the SV40 large T antigen (ATCC CRL-3216), BxPC-3 (ATCC CRL-1420; American Type Culture Collection, Manassas, VA) and MIA PaCa-2 (ATCC CRL-1687) are human pancreatic cancer cells. Tumor cells were cultured in DMEM, 1% GlutaMAX (Gibco, ThermoFisher), 100 IU/mL penicillin, 100 µg/mL streptomycin (Pan-Biotech), and 10% (v/v) heat-inactivated fetal calf serum (Sigma-Aldrich, St. Louis, USA).

### 2.2 CAR T cell generation

Cryopreserved PBMCs were thawed and stimulated on the same day with the anti-CD3 monoclonal antibody (mAb) OKT-3, the CD28 mAb 15E8 and IL-2 (1000 IU/mL). Recombinant IL-2 (200 IU/mL) was added on days 2, 3, and 4 after activation (IL-2 was just added without performing a complete medium exchange). Retroviral transduction was performed as previously described in detail (29). Viral particles were added on day +2 and day +3 after activation of PBMCs. Four days after activation (day +4), CAR T cells were enriched by labeling CAR T cells with a biotinylated goat F(ab´)2 anti-human IgG antibody (Southern Biotech, Birmingham, AL, USA) followed by purification with anti-biotin microbeads (Miltenyi Biotec, Bergisch Gladbach, Germany). Following a 24-hour culture period in IL-2 free medium, CAR T cells were used for *in vitro* assays. Untransduced cells were generated by activation of PBMCs and subsequent expansion with IL-2, but without retroviral transduction. The CEA-specific CAR BW431/26scFv-Fc-CD28-ζ expression cassette was previously published (30). The vectors encoding the CEA-specific CAR together with shRNAs either targeting IRF4 (BW431/26scFv-Fc-CD28-ζ-I1, BW431/26scFv-Fc-CD28-ζ-I2) or kanamycin (BW431/26scFv-Fc-CD28-ζ-K) were synthesized by GenScript Biotech (Piscatawy, N.J., USA). TRCN0000433892 was used to generate the shRNA for BW431/26scFv-Fc-CD28-ζ-I1. TRCN0000014764 was used to generate the shRNA for BW431/26scFv-Fc-CD28-ζ-I2. A well- characterized control shRNA targeting the kanamycin gene was used to generate BW431/26scFv-Fc-CD28-ζ-K (19). The shRNA constructs were embedded in a modified miR-30 scaffold as previously described and inserted at the 3’ end of the CAR construct (31, 32).

### 2.3 Flow cytometry

Cells were incubated with antibodies at 4°C for 15 min for surface staining. For intracellular staining, cells were fixed and permeabilized with the “Transcription Buffer” set (BD Biosciences, Franklin Lakes, NJ, USA) for 30 min at 4°C. For STAT5 staining, the “Transcription Factor Phospho Buffer Set” (BD Biosciences, Franklin Lakes, NJ, USA) was used according to the manufacturer´s instructions. The viability dye eFluor 780 (ThermoFisher, Waltham, MA, USA) was employed for live/dead discrimination. Fluorescent-minus-one (FMO) controls were used for gating. The goat F(ab’)2 anti-human IgG-PE antibody and the goat F(ab’)2 anti-human IgG-FITC antibody were purchased from SouthernBiotech to detect the CAR. The following antibodies were obtained from Miltenyi Biotech: FITC-conjugated anti-CD3 (clone BW 264/56), FITC-conjugated anti-CD8 (clone BW135/80), APC-conjugated anti-CD4 (clone VIT4), PE-conjugated anti-CD25 (clone 4E3), APC-conjugated anti-CD70 (clone REA292), and APC Vio770-conjugated anti-CD66abcde (clone TET2). The following antibodies were purchased from Biolegend (San Diego, CA, USA): PerCP-Cy5.5-conjugated anti-CD27 (clone M-T271), PerCP-Cy5.5-conjugated anti-TIGIT (clone A15153G), BV421-conjugated anti-CD3 (clone OKT3), PerCP-Cy5.5-conjugated anti-CD86 (IT2.2), PE-conjugated anti-41BBL (clone 5F4), PerCP-Cy5.5-conjugated anti-CD28, and PE-conjugated anti-IRF4 (clone IRF4.3E4) together with the corresponding isotype control antibody. The following antibodies were purchased from BD Biosciences: BV421-conjugated anti-TIM3 (clone 7D3), BV421-conjugated anti-CD62L (clone DREG-56), BV605-conjugated anti-CD45RO (clone UCHL1), BV421-conjugated anti-CD8 (clone RPA-T8), FITC-conjugated anti-CD80 (clone L307.4), BV421-conjuagted anti-CD137 (clone 4B4-1), and BV421-conjugated anti-pSTAT5 (clone 47/STAT5 pY694). Immunofluorescence was measured using a BD FACSLyric (BD Biosciences) equipped with FACSuite software (BD Biosciences). Data were analyzed using the FlowJo software version 10.7.1 Express 5 (BD Biosciences).

### 2.4 Western blot analysis

After a 24-hour co-culture period with BxPC-3 cells, untransduced T cells, CEA-28ζ-K (Ctrl) CAR T cells, and CEA-28ζ-I1 CAR T cells were lysed (3 x 10^6^ cells per condition) and lysates were electrophoresed by SDS-PAGE in 4–12% (w/v) Bis-Tris gels under reducing conditions, blotted and probed with the anti−IRF4 mAb (clone IRF4.3E4, BioLegend) at 1:200 and by the peroxidase-labeled anti-rat IgG1 antibody (Sigma-Aldrich) at 1:5,000 dilution. Membranes were stripped and re-probed with peroxidase-labeled anti−β-actin antibody (Santa Cruz Biotechnology) at 1:20,000 dilution. Bands were detected by chemoluminescence (ChemiDoc Imaging System, BioRad).

### 2.5 Cytokine secretion

Cancer cells were seeded in 96 well round-bottom plates (1 x 10^5^ cells/well) overnight, before addition of untransduced T cells or CAR T cells (1 x 10^5^ cells/well). Alternatively, the anti-idiotypic monoclonal antibody BW2064/36 was coated overnight on 96 well plates at the indicated concentrations before adding CAR T cells (1 x 10^5^ cells/well). After 48 hours of co-culture, IL-2 and IFN-γ in culture supernatants were detected by ELISA as previously described (33). TNF-α was detected using the “human TNF-α Standard ABTS ELISA Development Kit” (PeproTech, Cranbury, NJ, USA) according to the manufacturer´s instructions.

### 2.6 Cytotoxicity assay

CAR T cells (0.125-10 × 10^4^ cells/well) were co-cultivated for 72 hours in 96 well round bottom plates with target cells (each 1 × 10^4^ cells/well) at the indicated effector to target ratios. The XTT-based colorimetric assay employing the “Cell Proliferation Kit II” (Roche Diagnostics, Mannheim, Germany) was used to determine specific cytotoxicity. Viability of tumor cells was calculated as mean values of six wells containing only tumor cells subtracted by the mean background level of wells containing medium only. Formation of formazan due to the presence of T cells was determined from triplicate wells containing T cells in the same number as in the corresponding experimental wells. The percentage of viable tumor cells in experimental wells was calculated as follows: viability (%) = [OD(experimental wells - corresponding number of T cells)]/[OD(tumor cells without T cells - medium)] × 100. Cytotoxicity (%) was defined as 100 - viability (%).

### 2.7 Repetitive stimulation assay

BxPC-3 cells labeled with GFP were seeded in 12 well plates at 0.1 × 10^6^ cells per well. After 24 hours, 0.1 × 10^6^ CAR T cells were added per well. After three days (Round 1, R1), the wells were resuspended and harvested. Subsequently, the wells were trypsinized, resuspended with T cell medium, and pooled with the initial supernatant. Then, cells were washed with PBS and resuspended in 1 ml T cell medium. Finally, 100 μl were used for cell counting (live GFP^+^ tumor cells and live CD3^+^/CAR^+^ CAR T cells) *via* flow cytometry using counting beads (“CountBright”, ThermoFisher), and the remaining 900 μl were added to a new 12 well plate with 0.1 × 10^6^ BxPC-3 cells for four days (round 2, R2). The procedure was reiterated for round 3 (R3) and round 4 (R4). For flow cytometric analyses, unlabeled BxPC-3 cells were employed, and three rounds (R1-R3) of stimulation were performed.

### 2.8 CD107a degranulation assay

Degranulation of CAR T cells in response to BxPC-3 cells was measured using a conventional CD107a staining. BxPC-3 cells were seeded overnight in 96 well plates at 0.05 × 10^6^ cells per well. At the end of each round (R1-3) of stimulation, CEA-28ζ-K CAR T cells and CEA-28ζ-I1 CAR T cells were re-stimulated with BxPC-3 cells. Monensin (eBioscience, San Diego, CA, USA) at a final concentration of 1 µM and a FITC-conjugated anti-CD107a antibody (BD Biosciences, San Jose, CA, USA, clone: H4A3) were added at the beginning of co-culture. Four hours later, T cells were stained with the viability dye eFluor 780, the goat F(ab’)2 anti-human IgG-PE antibody, a BV421-conjugated anti-CD8 antibody (BD), and an APC-conjugated anti-CD4 antibody (Miltenyi Biotech). Subsequently, degranulation measured as CD107a^+^ cells was analyzed *via* flow cytometry.

### 2.9 Phycoerythrin QuantiBRITE antigen density quantitation assay

The density of CEA on the surface of 293T cells, BxPC3-cells and MIA PaCa-2 cells was determined using the QuantiBRITE PE assay (LOT: #60550, BD) according to the manufacturer´s instructions in conjunction with a PE-labeled anti-CEA antibody (clone TET2, Miltenyi) using flow cytometry. The assay is predicated on a pre-calibrated standard bead set involving known numbers of fluorophore molecules bound per bead to calibrate and translate the intensity of fluorescence signals obtained by flow cytometry into the number of fluorophores.

### 2.10 Statistical analysis

Statistical analysis was performed using GraphPad Prism, Version 9 (GraphPad Software, San Diego, CA, USA). P values were calculated by Student`s t test or paired t test as indicated; ns indicates not significant, * indicates p ≤ 0.05, ** indicates p ≤ 0.01, and *** indicates p ≤ 0.001.

## 3 Results

### 3.1 Generation of CAR T cells with reduced IRF4 expression

**Figure 1:**
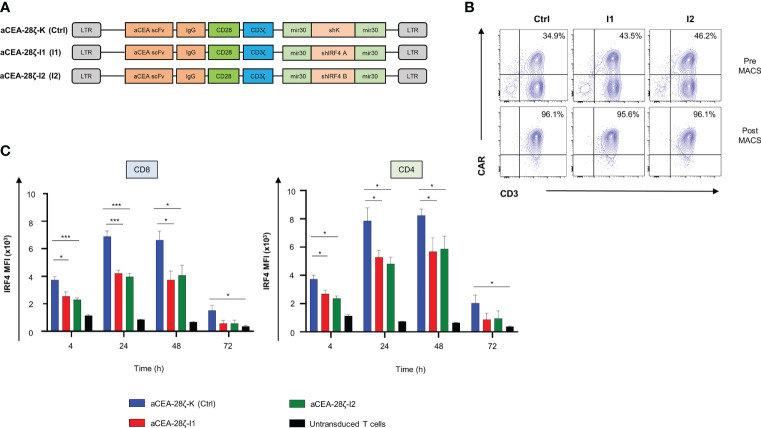
Downregulation of IRF4 expression in CAR T cells. **(A)** Schematic of CAR constructs. **(B)** CAR expression in T cells detected by staining with a phycoerythrin (PE)-labeled goat anti-human IgG antibody before (upper panels) and after (lower panels) magnetic cell separation (MACS). One representative donor out of four is shown. **(C)** Intracellular staining of IRF4 after stimulation with CEA^+^ BxPC-3 cells at the indicated time points in CD8^+^ (left panel) and CD4^+^ CAR T cells (right panel). CAR T cells were generated as described in the materials and methods section by activation of PBMCs followed by retroviral transduction. Untransduced cells were generated by activation of PBMCs and subsequent expansion with IL-2, but without retroviral transduction. Data represent means ± SEM of five donors, p values were calculated by Student´s t test, *indicates p ≤ 0.05, ***indicates p ≤ 0.001.

To generate CAR T cells with reduced IRF4 levels, we engineered a retroviral expression cassette coding for both a CAR (CEA-28ζ) specific for the carcinoembryonic antigen (CEA) and for an IRF4-specific shRNA embedded within the miRNA30 backbone to allow shRNA transcription by the CMV promotor within the LTR (**Figure 1A**). Two shRNAs targeting IRF4 were designed, CEA-28ζ-I1 and CEA-28ζ-I2. A vector with the well-established shRNA directed against the kanamycin gene (19) served as control construct (CEA-28ζ-K). After retroviral transduction CEA-28ζ-I1 CAR T cells and CEA-28ζ-I2 CAR T cells exhibited similar CAR expression levels (**Figure 1B**). CEA-28ζ-K CAR T cells displayed a slightly lower transduction rate (**Figure 1B**). CAR^+^ cells were enriched by magnetic-activated cell sorting (MACS) resulting in more than 90% CAR^+^ cells without substantial differences in CAR levels (**Figures 1B**, **S1A**). Enriched CAR T cell preparations were used in further analyses.

IRF4 is physiologically strongly upregulated after T cell activation (**Figure S1B**). To verify downregulation in the presence of shRNA, the IRF4 expression by CEA-specific CAR T cells was monitored up to 72 hours after co-culture with CEA^+^ BxPC-3 pancreatic cells. Upon CAR stimulation, IRF4 was upregulated after four hours in both CD8^+^ and CD4^+^ CAR T cells with IRF4 expression reaching a peak at 24-48 hours followed by a decline to baseline after 72 hours (**Figure 1C**, **Supplementary Figure 1C**). In comparison to control CEA-28ζ-K CAR T cells, CAR T cells transduced with the IRF4-specific shRNA showed a significantly reduced IRF4 expression at all time points. Both IRF4 shRNA constructs were similarly efficacious in reducing IRF4 expression. In further analyses, we used the CEA-28ζ-I1 construct. Prior to functional evaluation, we corroborated downregulation of IRF4 in T cells transduced with CEA-28ζ-I1 by Western blot analysis (**Supplementary Figure 1D**). Taken together, the one-vector retroviral system enabled downregulation of IRF4 by shRNA along with constitutive CAR expression which will facilitate convenient manufacturing of the respective CAR T cells.

### 3.2 IRF4 downregulation does not impair cytotoxicity of CAR T cells

**Figure 2:**
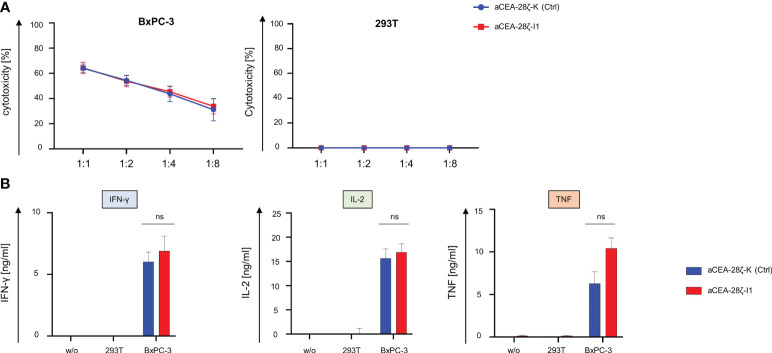
Cytotoxicity and cytokine signaling after IRF4 downregulation. **(A)** Cytotoxicity of CAR T cells upon a 24-hour co-culture with CEA^+^ BxPC-3 cells (left panel) and CEA^-^ 293T cells (right panel) was measured at the indicated effector to target ratios by an XTT-based colorimetric assay. Data represent means ± SEM of six donors, p values were calculated by Student´s t test. **(B)** ELISA-based quantification of CAR activation induced IFN-γ (left panel), IL-2 (middle panel), and TNF-α (right panel) in the supernatant after a 48-hour co-culture with medium (w/o), 293T cells, and BxPC-3 cells. Please note the different scales (pg/ml as compared to ng/ml in **Figure 2B**). Data represent means ± SEM of at least five donors, p values were calculated by Student´s t test, ns indicates not significant.

We recorded CAR-triggered cytotoxicity and cytokine secretion of CEA-28ζ-I1 CAR T cells with downregulated IRF4 in comparison to control CEA-28ζ-K CAR T cells. To this end, CAR T cells were co-cultured with CEA^-^ 293T cells and CEA^+^ BxPC-3 pancreatic cells for 24 hours. Across different effector to target cell ratios, both CEA-28ζ-I1 and CEA-28ζ-K CAR T cells were equally effective in killing of BxPC-3 cells (**Figure 2A**). No significant background cytotoxicity against 293T cells was recorded (**Figure 2B**). Furthermore, we interrogated cytokine secretion after 48 hours of co-culture with target cells using ELISA. Upon antigen-triggered stimulation with BxPC-3 cells for 48 hours, CEA-28ζ-I1 and CEA-28ζ-K secreted similar amounts of IL-2, IFN-γ and TNF-α (**Figure 2B**). Little spontaneous background cytokine release was detected (**Figure 2B**). Moreover, intracellular staining of IRF4 in untransduced T cells and CAR T cells was performed to assess baseline IRF4 expression at the start of *in vitro* assays. Similar to IRF4 expression after antigen-specific stimulation, baseline IRF4 levels were significantly reduced in CEA-28ζ-I1 CAR T cells as compared to CEA-28ζ-K CAR T cells (**Supplementary Figure 2**). In aggregate, IRF4 downregulation in CAR T cells did not impair basic cytotoxicity and cytokine release upon activation.

### 3.3 Downregulation of IRF4 enhances CAR T cell functionality under repetitive stimulatory conditions

**Figure 3:**
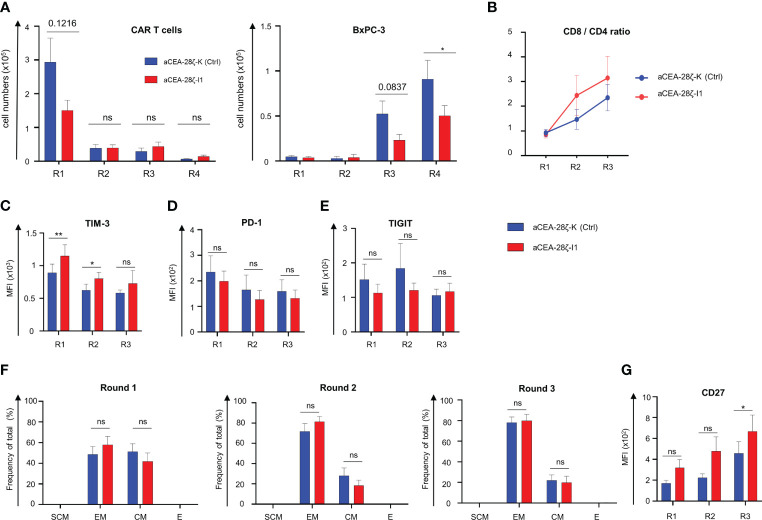
Downregulation of IRF4 enhances CAR T cell functionality. **(A)** CAR T cells (starting with 1 × 10^5^ CAR T cells) underwent four rounds (R1-R4) of stimulation with GFP-labeled CEA^+^ BxPC-3 cells (1 × 10^5^ tumor cells at the beginning of each round). At the end of each round, CAR T cells (live CD3^+^ CAR^+^) (left panel) and BxPC-3 cells (right panel) were quantified by flow cytometry. Data represent means ± SEM of six donors, p values were calculated by Student´s t test, ns indicates not significant, and * indicates p ≤ 0.05. **(B-G)** Phenotypic analysis of CD8^+^ CAR T cells during repetitive antigen stimulation. CAR T cells underwent three rounds (R1-R3) of antigen-stimulation with unlabeled BxPC-3 cells. At the end of each round, the CD8/CD4 T cell ratio was determined **(B)**. CAR T cells were stained for CD8 and further characterized regarding TIM-3 **(C)**, PD-1 **(D)**, TIGIT **(E)** expression and effector-memory cell differentiation: SCM = T stem-cell-memory (CD45RO^+^ CD62L^+^), EM = effector-memory (CD45RO^+^ CD62L^-^), CM = central-memory (CD45RO^+^ CD62L^+^), E = effector (CD45RO^-^ CD62L^-^) **(F)**, and CD27 expression **(G)**. Data represent geometric means of ± SEM of at least four donors, p values were calculated by paired t test, ns indicates not significant, *indicates p ≤ 0.05, **indicates p ≤ 0.01.

We aimed to mitigate CAR T cell exhaustion in the long-term upon repetitive antigen challenge by reducing IRF4 levels in CAR T cells. To address the issue, we assayed CAR T cell effector functions during four consecutive rounds (Round 1–4) of stimulation with GFP-labeled CEA^+^ BxPC-3 cells with each round lasting for three to four days. CEA-28ζ-K CAR T cells with physiological IRF4 levels expanded within the first round of stimulation followed by a contraction phase during the following rounds (**Figure 3A**). Similarly, CAR T cells with reduced IRF4 expression displayed expansion followed by swift contraction without major difference to control CAR T cells. With respect to anti-tumor activity, both CEA-28ζ-K and CEA-28ζ-I1 CAR T cells evinced robust elimination of BxPC-3 cells in the first two rounds of cancer cell challenge while cancer cell elimination declined in round three. However, CAR T cells with reduced IRF4 levels were still capable of eliminating half of seeded BxPC-3 cells in round four whereas the cytotoxic capacity of CEA-28ζ-K control CAR T cells was nearly extinguished (**Figure 3A**). Data demonstrate that CAR T cells with reduced IRF4 levels retain anti-cancer cell activity under repetitive challenge conditions for a longer period than conventional CAR T cells.

To elucidate the underlying mechanism, we recorded the CD8/CD4 ratio of CAR T cells, the expression of markers associated with T cell exhaustion, the differentiation state, and the expression of CD27 during repetitive challenge with CEA^+^ BxPC-3 cells. Starting from a relatively even level of 1 to 1 (no normalization was performed) the CD8/CD4 T cell ratio increased in both CAR T cell sets during repetitive antigen stimulation while IRF4 downregulation favored a higher portion of CD8^+^ T cells in later rounds (**Figure 3B**). To investigate whether the improved anti-cancer activity displayed by CEA-28ζ-I1 CAR T cells during repetitive antigen-challenge could derive from the higher frequency of CD8^+^ T cells, CD107a degranulation with simultaneous detection of CD8^+^ CAR T cells and CD4^+^ CAR T cells was recorded upon a short-term re-stimulation with BxPC-3 cells at the end of each round 1-3 (R1-3). Generally, the degranulation capacity of CAR T cells declined during repetitive stimulation, with both CD8^+^ and CD4^+^ CEA-28ζ-I1 CAR T cells exhibiting higher degranulation than CEA-28ζ-K control CAR T cells at the end of round three (**Supplementary Figures 3A + B**). The results are in line with the data obtained from the re-challenge assay (**Figure 3A**) suggesting an additional improvement of cytotoxicity derived from IRF4 downregulation that is independent from elevated CD8^+^ CAR T cell frequencies. TIM-3 was expressed at higher levels in CEA-28ζ-I1 CAR T cells as compared to CEA-28ζ-K control CAR T cells during the first two rounds of cancer cell challenge. The upregulation of PD-1 and TIGIT was similar in CEA-28ζ-I1 CAR T cells and CEA-28ζ-K control CAR T cells (**Figures 3C-E**, **Supplementary Figures 3C-E**). Starting with a relatively similar distribution of CD45RO^+^ CD62L^+^ central memory T cells and CD45RO^+^ and CD62L^-^ effector memory cells, both CAR T cells with and without IRF4 down-regulation shifted from central memory to effector memory T cells during the consecutive rounds of cancer cell challenge (**Figure 3F**, **Supplementary Figure 3F**). CEA-28ζ-I1 CAR T cells and control CAR T cells did not significantly differ in their effector-memory differentiation (**Figure 3F**, **Supplementary Figure 3F**). At the end of round three, the memory-associated molecule CD27 was substantially higher upregulated in CD8^+^ CEA-28ζ-I1 CAR T cells compared to control CAR T cells (**Figure 3G**). The effect was restricted to CD8^+^ T cells and was not recorded in CD4^+^ CEA-28ζ-I1 CAR T cells (**Supplementary Figure 3G**). Of note, CD27 upregulation in CD8^+^ CAR T cells was reported to be associated with enhanced CAR T cell functionality and sustained remissions in patients receiving CAR T cell therapy (34).

Next, we tracked the expression of the co-stimulatory molecules CD137 and CD28 during repetitive challenge. While CD137 was predominately upregulated in CD8^+^ CAR T cells during the first round of antigen challenge, CD4^+^ CEA-28ζ-I1 CAR T cells showed a significantly higher expression of CD137 as compared to CEA-28ζ-K control CAR T cells at end of the first and third round (**Supplementary Figure 4A**). As for both CD8^+^ and CD4^+^ T cells, the upregulation of CD28 was more pronounced in CEA-28ζ-K control CAR T cells as compared to CEA-28ζ-I1 CAR T cells at all time points (**Supplementary Figure 4B**). Finally, to investigate a potential transactivation of CAR T cells by tumor cells, we checked the expression of co-stimulatory ligands, such as CD70, 41BBL, CD80, and CD86, on BxPC-3 tumor cells by flow cytometry. None of these ligands were detected on BxPC-3 cells (**Supplementary Figure 4C**).

### 3.4 Downregulation of IRF4 in CAR T cells enables killing of target cells with low antigen density

**Figure 4:**
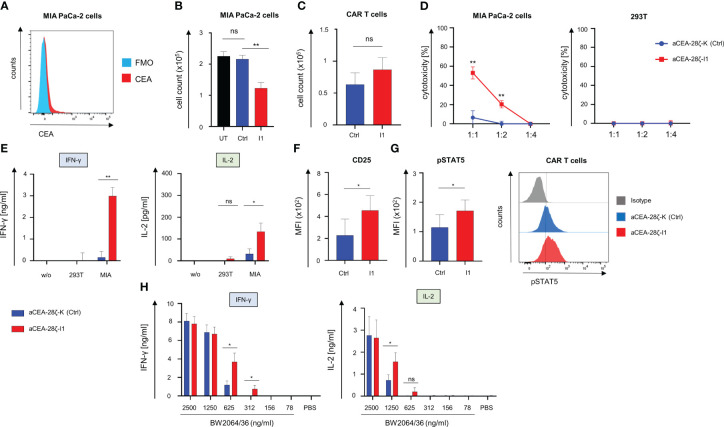
Downregulation of IRF4 in CAR T cells enables killing of targets with low antigen density. **(A)** Staining of target cells MIA PaCa-2 for CEA expression using an APC Vio 770-conjugated anti-CEA antibody. One representative staining out of three experiments is shown. **(B)** T cells (1 × 10^5^ T cells) with and without IRF4 downregulation were co-culture with GFP-labeled CEA^+^ MIA PaCa-2 cells (1 × 10^5^ tumor cells). After three days, CEA^+^ MIA PaCa-2 cells (live GFP^+^ cells) and CAR T cells (live CD3^+^ CAR^+^) **(C)** were counted by flow cytometry using counting beads. Data represent means ± SEM of five donors, p values were calculated by Student’s t test, ns: not significant, **p ≤ 0.01. **(D)** Cytotoxicity of CAR T cells upon a 72-hour co-culture with MIA PaCa-2 cells or CEA^-^ 293T cells was measured at the indicated effector to target cell ratios by an XTT-based colorimetric assay. Data represent means ± SEM of four donors, p values were calculated by Student´s t test. **(E)** IFN-γ and IL-2 in the supernatants after a 48-hour co-culture with medium (w/o), 293T cells, and MIA PaCa-2 cells was recorded by ELISA. Data represent means ± SEM of three donors, p values were calculated by Student´s t test, ns: not significant. **(F)** Staining of CAR T cells for CD25 and for pSTAT5 **(G)** after three days of co-culture with MIA-PaCa-2 cells. Data represent geometric means of ± SEM of three donors, p values were calculated by paired t test, ns indicates not significant, *indicates p ≤ 0.05. **(H)** ELISA-based quantification of CAR-activation induced IFN-γ and IL-2 in the supernatant after a 48-hour culture on 96 well. plates coated with the anti-idiotypic monoclonal antibody BW2064/36 at the indicated concentrations. Data represent means ± SEM of eight donors, p values were calculated by paired t test, *p ≤ 0.05.

Given the significant relevance of cancer cells with low antigen densities evading a CAR T cell attack, we next evaluated the impact of IRF4 downregulation on the killing of targets with low antigen density. To this end, we resorted to MIA PaCa-2 human pancreatic cancer cells that express CEA at low levels (35) (**Figure 4A**). Using these cells as targets, we compared the CAR-redirected cytotoxicity of CEA-28ζ-I1 CAR T cells with IRF4^low^ levels to CEA-28ζ-K CAR T cells with physiological IRF4 levels in a FACS-based 72-hour killing assay. GFP-labeled MIA PaCa-2 cells were co-cultured with CAR T cells at a 1 to 1 ratio. After 72 hours, the numbers of live GFP^+^ MIA PaCa-2 cells and CAR T cells (live CD3^+^ CAR^+^) were analyzed using counting beads. As summarized in **Figure 4B**, IRF4 downregulation augmented the CAR-triggered elimination of tumor cells with low antigen levels whereas CEA-28ζ-K control CAR T cells did not exhibit significant cytotoxicity towards GFP-labeled MIA PaCa-2 cells. During this period, no substantial expansion of CAR T cells occurred, and absolute CAR T cell numbers did not differ significantly (**Figure 4C**). In order to confirm the enhanced cytotoxicity of CEA-28ζ-I1 CAR T cells against MIA PaCa-2 cells, a 72-hour XTT-based colorimetric killing assay employing different effector to target ratios was conducted (**Figure 4D**). No antigen-independent cytotoxicity against CEA^-^ 293T cells was observed in this assay corroborating an increase of CEA-specific cytotoxicity without raising overall unspecific cytotoxicity.

To address the mechanism of improved functional capacities, we recorded the CAR-triggered cytokine release upon engagement of target cells. CEA-28ζ-I1 CAR T cells with reduced IRF4 levels secreted significantly higher quantities of IFN-γ and IL-2 as compared to CEA-28ζ-K control CAR T cells (**Figure 4E**). High secretion of cytokine levels went along with increased levels of the IL-2 receptor (CD25) on IRF4^low^ CAR T cells after a three-day co-culture with MIA PaCa-2 cells. (**Figure 4F**). In addition, the downstream signaling mediator phospho-STAT5 is increased in CEA-28ζ-I1 CAR T cells (**Figure 4G**) reflecting a higher level of activation in response to target cells with low antigen-expression. To further elucidate the sensitivity of CEA-28ζ-I1 CAR T cells, we flow-cytometrically determined the antigen densities of target cells *via* the QuantiBRITE phycoerythrin (PE) assay in conjunction with a PE-labeled anti-CEA antibody. While CEA^high^ BxPC-3 cells expressed an average of 282735 CEA molecules per cell, CEA^low^ MIA PaCa-2 cells showed a more than 100-fold lower CEA expression averaging 602 CEA molecules per cell (**Supplementary Figure 5**).

To address whether IRF4 downregulation improves sensitivity to antigen by CAR T cells, we stimulated CAR T cells with decrementing concentrations of the anti-idiotypic monoclonal antibody BW2064/36, which acts as surrogate antigen for CEA (36), to provide antigen-specific stimulation through the CEA-specific CAR. Response to CAR-triggered T cell activation was determined by reading IFN-γ and IL-2 release during a 48-hour stimulation period. While both CEA-28ζ-K control CAR T cells and CEA-28ζ-I1 CAR T cells secreted similar amounts of cytokines at high antigen concentrations, CEA-28ζ-I1 CAR T cells with low IRF4 levels released significantly higher amounts of cytokines at low antigen concentrations (**Figure 4H**). We concluded that IRF4 downregulation resulted in enhanced antigen-sensitivity for CAR T cell activation.

## 4 Discussion

Evidence indicates that IRF4 plays a crucial role in establishing and maintaining T cell exhaustion during chronic infection (26). To extend a CAR T cell anti-cancer cell response upon repetitive antigen encounter, we reduced IRF4 levels by expressing an IRF4-specific shRNA in CAR T cells. For convenient manufacturing, we designed a retroviral vector that encodes for both the shRNA module targeting the IRF4 gene and the expression of the CEA-specific CAR. The vector allows transduction of T cells to similar rates as canonical vectors encoding exclusively the CAR. ShRNA-mediated IRF4 downregulation did not impair primary cytotoxicity and cytokine secretion of engineered CAR T cells in short-term assays. However, under conditions of repetitive challenge with cognate cancer cells, CEA-28ζ-I1 CAR T cells with IRF4 downregulation showed superior cancer cell elimination compared with conventional CAR T cells. Remarkably, CD8^+^ CEA-28ζ-I1 CAR T cells with reduced IRF4 levels upregulated CD27 throughout repetitive antigen challenge. Improved CD27 expression has been linked to superior CAR T cell functionality and response to therapy (34). Transcriptome profiling revealed that memory-related genes were enriched in patients attaining complete responses while gene signatures involved in effector cell differentiation, glycolysis, exhaustion, and apoptosis were selectively upregulated in non-responding patients (34). Remarkably, long-lasting complete remissions were associated with enhanced CD27 expression in circulating CD8^+^ T cells prior to CAR T cell manufacturing. Physiologically, increased CD27 is linked to enhanced T cell functional capacities, survival, and memory formation (37). In sum, we conclude that elevated CD27 expression in CAR T cells may reflect a status of augmented functionality of CEA-28ζ-I1 CAR T cells during repetitive antigen stimulation. In accordance with improved CD27 levels, we did not record major indications for exhaustion since PD1 and TIGIT were not altered. TIM-3 declined after initial increase in early phases of stimulation.

Our analyses also highlight that IRF4 downregulation results in improved antigen sensitivity of CAR T cells to cognate target cells, i.e., decrease in antigen threshold for activation. This enables in successful targeting of antigen-low cells that are neglected by conventional CAR T cells. We assume that this is due to an elevated T cell activation state in response to CD25 and phospho-STAT5 upregulation which both pertain to the IL-2 pathway. Recently, c-Jun overexpression has been shown to eventuate in enhanced antigen-sensitivity by directly augmenting c-Jun-mediated transcriptional activation of target genes, such as IL-2, through displacing AP1-IRF4 complexes from chromatin. AP1-IRF4 complexes induce an exhaustion-related transcriptional program that blunts T cell sensitivity to antigen (9). In the present study, we also observed an elevated activity of the IL-2 pathway in response to IRF4 downregulation, which might also originate from a reduction in chromatin-bound AP-1–IRF4 complexes. Consequently, the enhanced transcriptional access to key T cell effector genes, such as IL-2, might in turn result in enhanced T cell activity particularly visible under conditions of low-antigen density. Nevertheless, extensive chromatin analyses using ATAC-sequencing are required in follow-up studies to thoroughly examine this hypothesis.

Improving antigen sensitivity of CAR T cells has substantial relevance, since tumor evasion of immune cell recognition by low target antigen levels is a leading cause for relapse after CAR T cell therapy (5). Previous strategies to target tumor cells with low antigen density encompass an alternative CAR design, the use of HLA-independent T cell receptors (38) or the co-expression of c-Jun (9). While sensing cancer cells with low antigen levels is beneficial in avoiding tumor relapse, augmenting antigen sensitivity of CAR T cells may raise the risk for on-target/off-tumor toxicities necessitating a careful selection of target antigens with no or limited expression on healthy tissues.

Although the cytokine secretion of conventional CAR T cells and CAR T cells with IRF4 downregulation was not significantly different in our study, the higher activation level of CAR T cells with IRF4 downregulation warrants an enhanced vigilance for cytokine release syndrome (CRS). Moreover, the secretion of central drivers of CRS, such as IL-6 and IL-1β, needs to be determined in future studies. Another potentially lethal side-effect of CAR T cell therapy is constituted by the immune effector cell-associated neurotoxicity syndrome (ICANS). Contrary to CRS, the mechanistic basis for the occurrence of ICANS is still largely unclear and requires further elucidation (39). In a recent study relying on single-cell RNA sequencing data to identify potential on-target off-tumor expression of the CAR antigen CD19, investigators found low-level expression of CD19 in brain mural cells posing a potential on-target mechanism for neurotoxicity in CD19-directed CAR T cell therapy (40). Against the backdrop of this finding, CD19-specific CAR T cells with enhanced antigen sensitivity should be tightly monitored for potentially causing neurotoxicity. Nevertheless, the majority of patients undergoing CD19-directed CAR T cell therapy do not experience neurotoxicity, and cerebral CD19 expression is not uniform across patients. With the selection of target antigens with no or limited expression on healthy tissues being the most critical step to avoid potentially lethal on-target/off-tumor toxicities (41), suicide genes (42) and initial infusions of CAR T cells with transient CAR expression generated by RNA electroporation should be considered to counteract toxicities (43). For the clinical translation of CAR T cells with enhanced antigen sensitivity, a crucial prerequisite is posed by the selection of safe antigens, the distribution of which has to be checked at the RNA and protein level in all available tissues. Moreover, animal models with orthotopic expression of human antigens, such as exemplified by mice expressing the human mesothelin protein in the lung (44), could be exploited to assess the *in vivo* antigen sensitivity of CAR T cells and the risk for on-target off-tumor toxicity.

In a recent study, IRF4 emerged as crucial regulator of CAR T cell exhaustion upon repetitive encounter of cancer cells (45). Single-cell ATAC-seq analyses identified regulatory networks driving CAR T cell exhaustion with IRF4 as one of the potentially crucial factors. Correspondingly, shRNA-mediated knockdown of IRF4 counteracted exhaustion, inhibited T cell differentiation, improved CAR T cell cytotoxicity, and augmented CAR T cell performance in leukemia bearing mice (45). However, during three rounds of cancer cell challenges we did not detect loss of functional capacities or major differences in effector-memory phenotypes or the expression of exhaustion markers, except transient upregulation of TIM-3, along with IRF4 downregulation. In contrast, we observed augmented anti-cancer cell capacities of CAR T cells after several rounds of repetitive antigen stimulation.

During repetitive antigen challenge we observed phenotypic changes induced by IRF4 downregulation that differed between CD8^+^ and CD4^+^ CAR T cells. Most notably, CD27 upregulation was highest in CD8^+^ CAR T cells with IRF4 downregulation, whereas CD27 upregulation was not significantly different in CD4^+^ CAR T cells. In T cells, CD27 co-stimulation is known to augment survival and anti-tumor activity (37). In a previous study investigating the impact of IRF4 downregulation on the functionality and expansion of CD8^+^ T cells during acute viral infections, CD8^+^ T cells with downregulated IRF4 levels exhibited CD27 upregulation and superior functionality as compared to wild type T cells (46). Especially in CD8^+^ T cells, direct transcriptional actions of IRF4 on CD27 gene expression could be hypothesized. The preferential upregulation of the survival-promoting molecule CD27 on CD8^+^ T cells might contribute to the increase in the CD8/CD4 ratio during repetitive antigen stimulation. Published data indicate a role for IRF4 as driver of T cell differentiation (26). Correspondingly, we observed a delayed effector cell differentiation and greater preservation of a central memory phenotype in CD4^+^ CAR T cells following IRF4 downregulation. Unexpectedly, IRF4 downregulation did not impact effector differentiation in CD8^+^ CAR T cells during repetitive antigen stimulation which might require even lower IRF4 levels. In contrast to CD27, the expression of CD28 was consistently lower in CAR T cells with IRF4 downregulation and generally higher in CD4^+^ CAR T cells during the course of repetitive antigen challenge. So far, no reports investigating the connection between IRF4 expression and CD28 expression in T cells have been published. Our data suggest that CD28 is a potential transcriptional target of IRF4.

Taken together, our data highlight downregulation of IRF4 in CAR T cells as a potent tool to augment functional capacities and antigen sensitivity of CAR T cells. Delivery of the IRF4-specific shRNA together with the CAR expression cassette by a one-vector system makes efficient manufacturing of CAR T cells in a GMP conform process feasible suggesting translation to clinical application.

## Data availability statement

The raw data supporting the conclusions of this article will be made available by the authors, without undue reservation.

## Ethics statement

The studies involving human participants were reviewed and approved by Institutional Review Board of the University of Regensburg (21-2224-101 Regensburg). The patients/participants provided their written informed consent to participate in this study.

## Author contributions

The concept of the study was designed by DH, WH, and HA. Experimental design was done by DH, VB, JH, and HA. Experiments were performed by DH. DH and HA wrote the manuscript. The manuscript was reviewed by all co-authors. All authors contributed to the article and approved the submitted version.

## Supplementary material

Supplementary Figure 1
**(A-D)** CAR T cells were generated as described in the materials and methods section by activation of PBMCs followed by retroviral transduction. Untransduced cells were generated by activation of PBMCs and subsequent expansion with IL-2, but without retroviral transduction. **(A)** Percentage of CAR expressing cells after MACS enrichment (left panel) and CAR MFI after MACS enrichment (right panel). Data represent means ± SEM of four donors, p values were calculated by Student´s t test, ns: not significant. **(B)** Intracellular staining of IRF4 after stimulation of untransduced T cells or CEA-28ζ-K control CAR T cells with coated anti-CD3 mAb OKT-3 (2.5 μg/ml) and anti-CD28 mAb 15E8 (5 μg/ml) after 24 hours in CD8^+^ (left panel) and CD4^+^ T cells (right panel). Please note the stimulation with anti-CD3 mAb OKT-3 and anti-CD28 mAb was performed in addition to the standard activation and the transduction procedure. One representative donor out of five donors is shown. **(C)** Histograms showing the intracellular staining of IRF4 in CD8^+^ (upper panels) and CD4^+^ CAR T cells (lower panels) after stimulation with CEA^+^ BxPC-3 cells at the indicated time points. The values for mean fluorescent intensity (MFI) of IRF4-PE staining are depicted in the histograms. One representative donor out of five donors is shown. **(D)** Western blot showing IRF4 protein expression in untransduced (UT) T cells, CEA-28ζ-K (Ctrl) CAR T cells, and CEA-28ζ-I1 CAR T cells after a 24-hour co-culture period with BxPC-3 cells. One representative donor out of three donors is shown.

Supplementary Figure 2 Intracellular staining of IRF4 in untransduced T cells and CAR T cells after a 24-hour culture period in IL-2 free medium to assess IRF4 expression at the start of *in-vitro* assays. CAR T cells were generated as described in the materials and methods section by activation of PBMCs followed by retroviral transduction. Untransduced cells were generated by activation of PBMCs and subsequent expansion with IL-2, but without retroviral transduction. Data represent means ± SEM of four donors, p values were calculated by Student´s t test, ns indicates not significant, and ** indicates p ≤ 0.01. Additionally, representative histograms are shown (right panel) with values for mean fluorescent intensity of IRF4-PE staining embedded within the histograms. One representative donor out of four donors is shown.

Supplementary Figure 3
**(A+B)** CD107a degranulation assay to separately evaluate the cytotoxic potential of CD8^+^ and CD4^+^ CAR T cells during repetitive stimulation using unlabeled BxPC-3 cells. At the end of each round (R1-3), CAR T cells were re-stimulated with unlabeled BxPC-3 cells. After four hours, surface expression of CD107a was measured separately for CD8^+^ and CD4^+^ CAR T cells *via* flow cytometry. **(A)** Data represent means of ± SEM of four donors, p values were calculated by Student´s t test, ns: not significant, * p ≤ 0.05, and ** p ≤ 0.01. **(B)** Representative dot plots showing degranulation in CD8^+^ (left panels) and CD4^+^ (right panels) CAR T cells at the end of round three. One representative donor out of four donors is shown. **(C-G)** Phenotypic analysis of CD4^+^ CAR T cells during repetitive antigen stimulation. CAR T cells underwent three rounds (R1-R3) of antigen stimulation with unlabeled BxPC-3 cells. At the end of each round, CAR T cells were stained for CD4 and further characterized with respect to TIM-3 **(C)**, PD-1 **(D)**, TIGIT expression **(E)**, and effector-memory cell differentiation: SCM = T stem-cell-memory (CD45RO^+^ CD62L^+^), EM = effector-memory (CD45RO^+^ CD62L^-^), CM = central-memory (CD45RO^+^ CD62L^+^), E = effector (CD45RO^-^ CD62L^-^) **(F)**, and CD27 expression **(G)**. Data represent geometric means of ± SEM of at least four donors, p values were calculated by paired t test, ns: not significant, * p ≤ 0.05.

Supplementary Figure 4
**(A+B)** Phenotypic analysis of CAR T cells during repetitive antigen stimulation. CAR T cells underwent three rounds (R1-R3) of antigen-stimulation with unlabeled BxPC-3 cells. At the end of each round, CAR T cells were stained for CD8 as well as CD4, and further characterized regarding CD137 **(A)** and CD28 **(B)** expression. Data represent geometric means of ± SEM of four donors, p values were calculated by paired t test, ns indicates not significant, * indicates p ≤ 0.05, ** indicates p ≤ 0.01. **(C)** Staining of target cells BxPC-3 for the expression of co-stimulatory ligands using an APC -conjugated anti-CD70 antibody, a PE-conjugated anti-41BBL antibody, a FITC-conjugated anti-CD80 antibody, and a PerCPCy5.5-conjugated anti-CD86 antibody. Fluorescent-minus-one (FMO) were used as controls. One representative staining out of three experiments is shown.

Supplementary Figure 5 Antigen densities of CEA^-^ 293T cells, CEA^low^ MIA PaCa-2 cells and CEA^high^ BxPC-3 cells as determined *via* QuantiBRITE phycoerythrin (PE) assay in conjunction with a PE-labeled anti-CEA antibody using flow cytometry. Data represent means (shown on top of bars) ± SEM of three independent experiments.
