# Supplementary figures and images for: IRF4 downregulation improves sensitivity and endurance of CAR T cell functional capacities
Source: Front Immunol. 2023 May 23;14:1185618. doi: 10.3389/fimmu.2023.1185618 (PMC10243527; doi:10.3389/fimmu.2023.1185618)

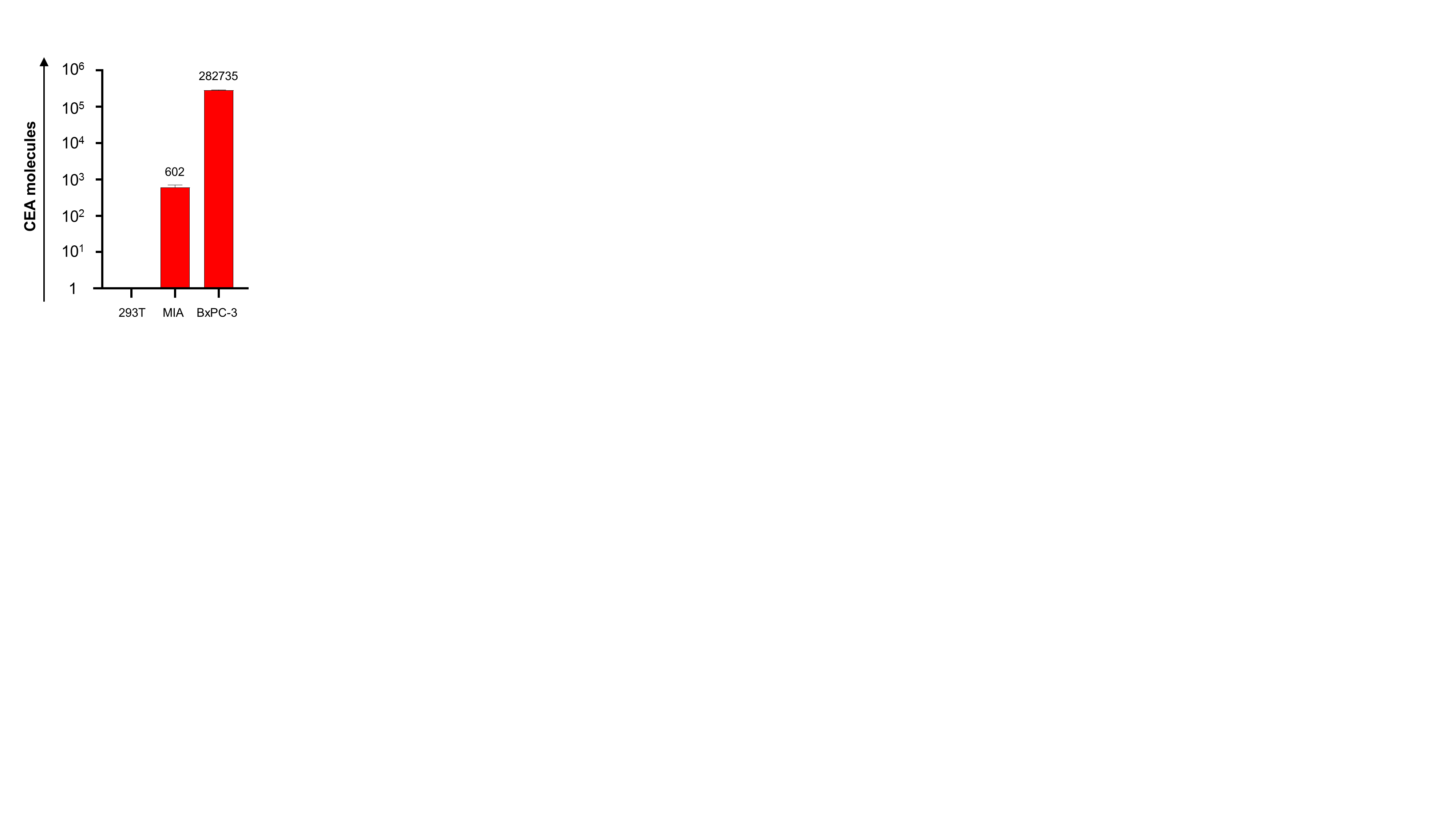

Supplement: Supplementary Figure 1 — (A-D) CAR T cells were generated as described in the materials and methods section by activation of PBMCs followed by retroviral transduction. Untransduced cells were generated by activation of PBMCs and subsequent expansion with IL-2, but without retroviral transduction. (A) Percentage of CAR expressing cells after MACS enrichment (left panel) and CAR MFI after MACS enrichment (right panel). Data represent means ± SEM of four donors, p values were calculated by Student´s t test, ns: not significant. (B) Intracellular staining of IRF4 after stimulation of untransduced T cells or CEA-28ζ-K control CAR T cells with coated anti-CD3 mAb OKT-3 (2.5 μg/ml) and anti-CD28 mAb 15E8 (5 μg/ml) after 24 hours in CD8+ (left panel) and CD4+ T cells (right panel). Please note the stimulation with anti-CD3 mAb OKT-3 and anti-CD28 mAb was performed in addition to the standard activation and the transduction procedure. One representative donor out of five donors is shown. (C) Histograms showing the intracellular staining of IRF4 in CD8+ (upper panels) and CD4+ CAR T cells (lower panels) after stimulation with CEA+ BxPC-3 cells at the indicated time points. The values for mean fluorescent intensity (MFI) of IRF4-PE staining are depicted in the histograms. One representative donor out of five donors is shown. (D) Western blot showing IRF4 protein expression in untransduced (UT) T cells, CEA-28ζ-K (Ctrl) CAR T cells, and CEA-28ζ-I1 CAR T cells after a 24-hour co-culture period with BxPC-3 cells. One representative donor out of three donors is shown. [file Image_1.tif]

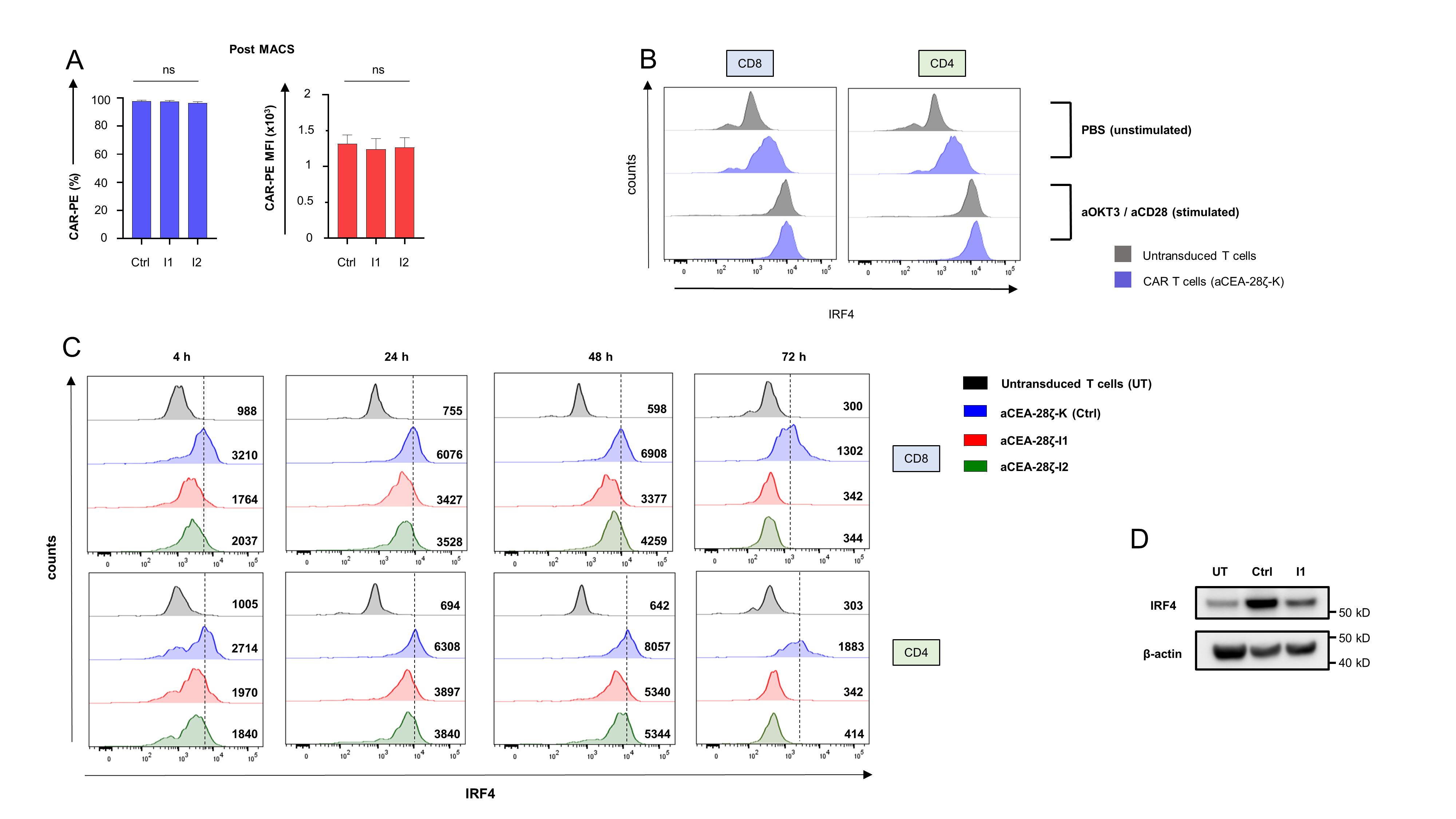

Supplement: Supplementary Figure 2 — Intracellular staining of IRF4 in untransduced T cells and CAR T cells after a 24-hour culture period in IL-2 free medium to assess IRF4 expression at the start of in-vitro assays. CAR T cells were generated as described in the materials and methods section by activation of PBMCs followed by retroviral transduction. Untransduced cells were generated by activation of PBMCs and subsequent expansion with IL-2, but without retroviral transduction. Data represent means ± SEM of four donors, p values were calculated by Student´s t test, ns indicates not significant, and ** indicates p ≤ 0.01. Additionally, representative histograms are shown (right panel) with values for mean fluorescent intensity of IRF4-PE staining embedded within the histograms. One representative donor out of four donors is shown. [file Image_2.tif]

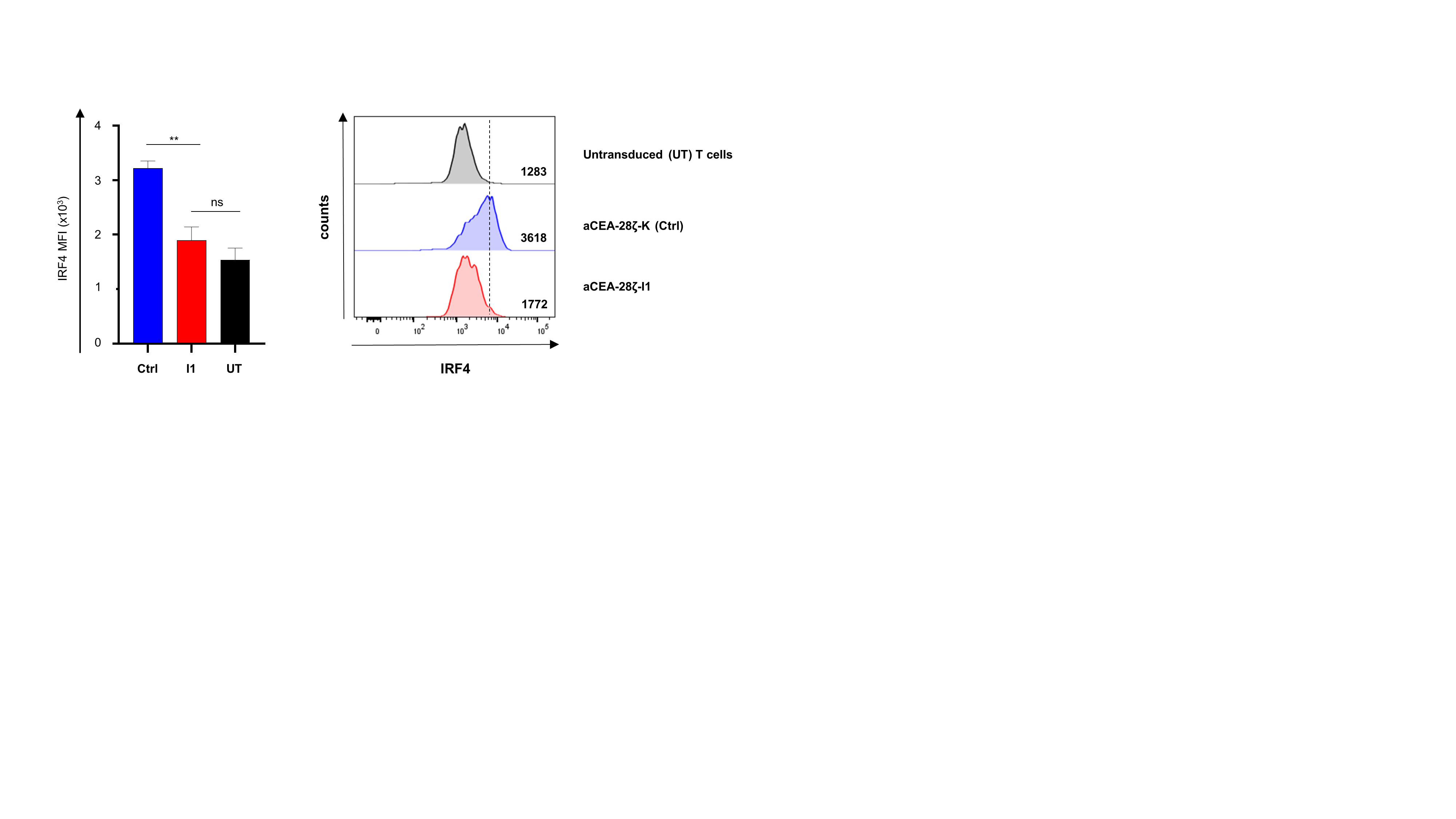

Supplement: Supplementary Figure 3 — (A+B) CD107a degranulation assay to separately evaluate the cytotoxic potential of CD8+ and CD4+ CAR T cells during repetitive stimulation using unlabeled BxPC-3 cells. At the end of each round (R1-3), CAR T cells were re-stimulated with unlabeled BxPC-3 cells. After four hours, surface expression of CD107a was measured separately for CD8+ and CD4+ CAR T cells via flow cytometry. (A) Data represent means of ± SEM of four donors, p values were calculated by Student´s t test, ns: not significant, * p ≤ 0.05, and ** p ≤ 0.01. (B) Representative dot plots showing degranulation in CD8+ (left panels) and CD4+ (right panels) CAR T cells at the end of round three. One representative donor out of four donors is shown. (C-G) Phenotypic analysis of CD4+ CAR T cells during repetitive antigen stimulation. CAR T cells underwent three rounds (R1-R3) of antigen stimulation with unlabeled BxPC-3 cells. At the end of each round, CAR T cells were stained for CD4 and further characterized with respect to TIM-3 (C), PD-1 (D), TIGIT expression (E), and effector-memory cell differentiation: SCM = T stem-cell-memory (CD45RO+ CD62L+), EM = effector-memory (CD45RO+ CD62L-), CM = central-memory (CD45RO+ CD62L+), E = effector (CD45RO- CD62L-) (F), and CD27 expression (G). Data represent geometric means of ± SEM of at least four donors, p values were calculated by paired t test, ns: not significant, * p ≤ 0.05. [file Image_3.tif]

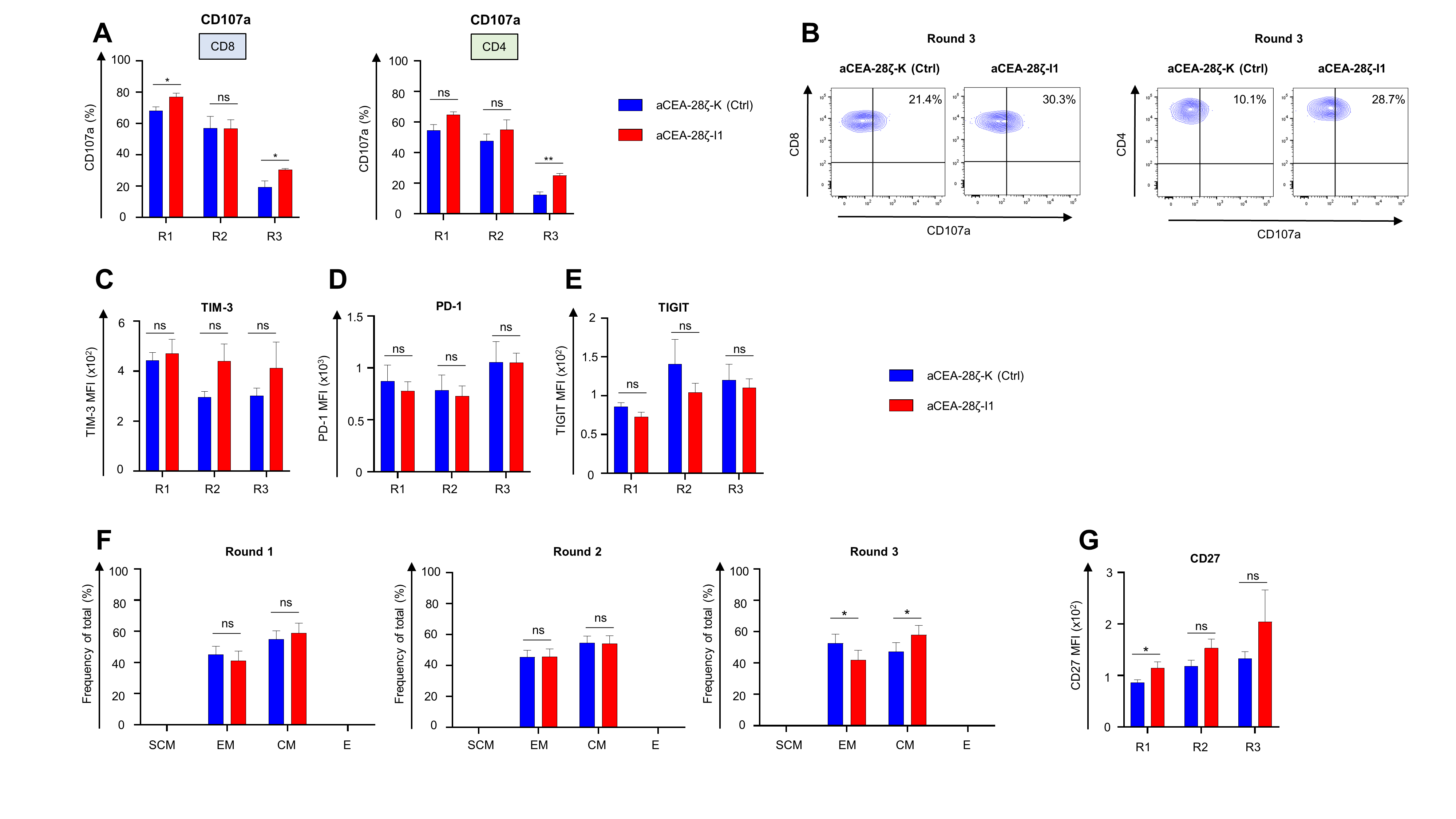

Supplement: Supplementary Figure 4 — (A+B) Phenotypic analysis of CAR T cells during repetitive antigen stimulation. CAR T cells underwent three rounds (R1-R3) of antigen-stimulation with unlabeled BxPC-3 cells. At the end of each round, CAR T cells were stained for CD8 as well as CD4, and further characterized regarding CD137 (A) and CD28 (B) expression. Data represent geometric means of ± SEM of four donors, p values were calculated by paired t test, ns indicates not significant, * indicates p ≤ 0.05, ** indicates p ≤ 0.01. (C) Staining of target cells BxPC-3 for the expression of co-stimulatory ligands using an APC -conjugated anti-CD70 antibody, a PE-conjugated anti-41BBL antibody, a FITC-conjugated anti-CD80 antibody, and a PerCPCy5.5-conjugated anti-CD86 antibody. Fluorescent-minus-one (FMO) were used as controls. One representative staining out of three experiments is shown. [file Image_4.tif]

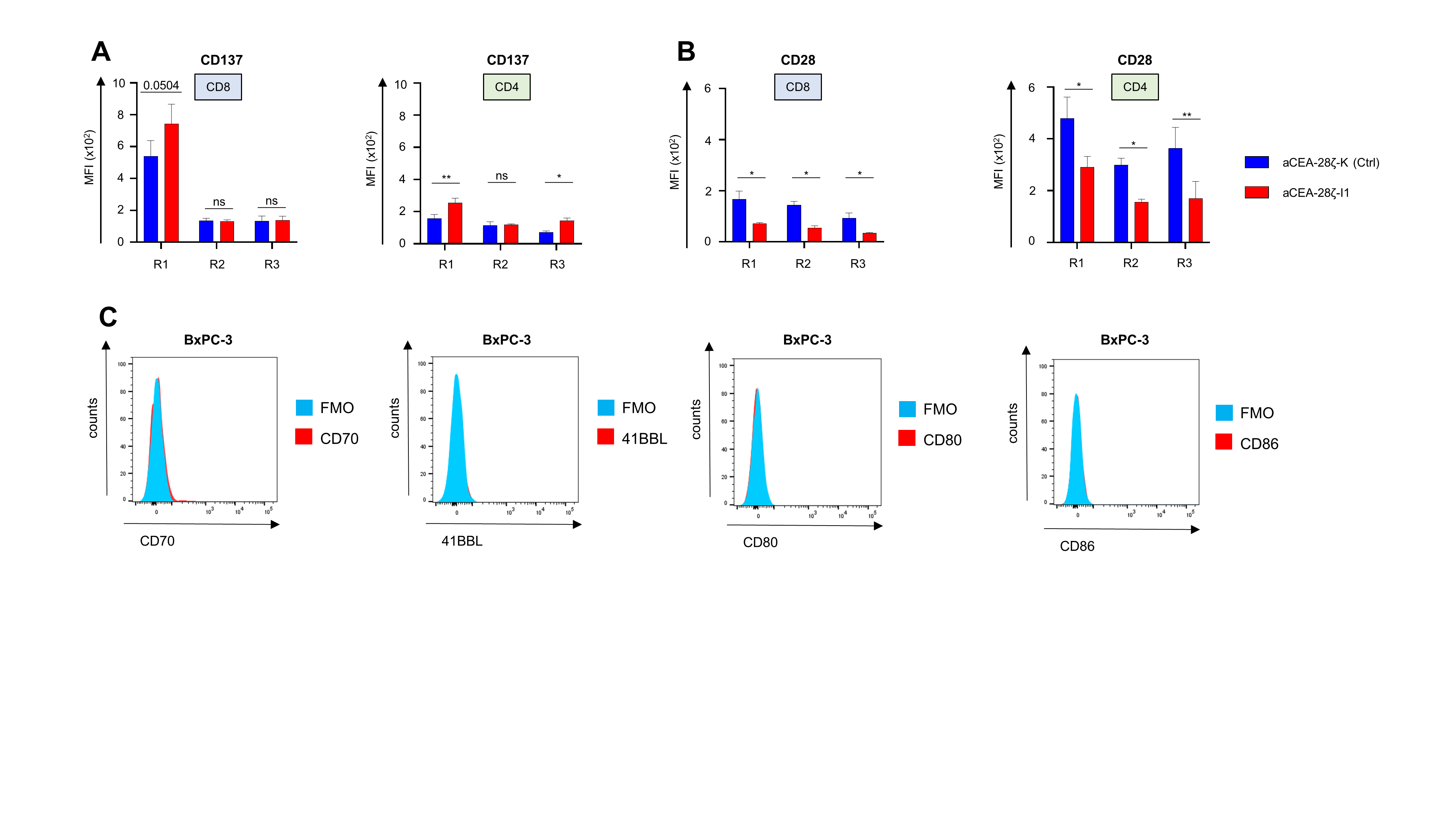

Supplement: Supplementary Figure 5 — Antigen densities of CEA- 293T cells, CEAlow MIA PaCa-2 cells and CEAhigh BxPC-3 cells as determined via QuantiBRITE phycoerythrin (PE) assay in conjunction with a PE-labeled anti-CEA antibody using flow cytometry. Data represent means (shown on top of bars) ± SEM of three independent experiments. [file Image_5.tif]
